# MambaGLA-NPC: a 3D framework integrating CNN and state space models for robust multi-center nasopharyngeal carcinoma segmentation

**DOI:** 10.3389/fgeed.2026.1954329

**Published:** 2026-09-29

**Authors:** Wei Jin, Xiaobo Chen, Weifeng Jin, Hai Liao, Li Yu

**Affiliations:** 1 School of Artificial Intelligence for Traditional Chinese Medicine, Zhejiang Chinese Medical University, Hangzhou, Zhejiang, China; 2 Department of Radiology, Guangdong Provincial People’s Hospital (Guangdong Academy of Medical Sciences), Southern Medical University, Guangzhou, Guangdong, China; 3 Guangdong Provincial Key Laboratory of Artificial Intelligence in Medical Image Analysis and Application, Guangzhou, Guangdong, China; 4 School of Pharmaceutical Sciences, Zhejiang Chinese Medical University, Hangzhou, Zhejiang, China; 5 Department of Medical Imaging Center, Guangxi Medical University Cancer Hospital, Guangxi Clinical Research Center for Medical Imaging Construction, Guangxi Key Clinical Specialty (Medical Imaging Department), Dominant Cultivation Discipline of Guangxi Medical University Cancer Hospital (Medical Imaging Department), Nanning, China; 6 School of Basic Medical Sciences, Zhejiang Chinese Medical University, Hangzhou, Zhejiang, China

**Keywords:** 3D segmentation, mamba, multi-center study, nasopharyngeal carcinoma, state space model

## Abstract

**Background and Objective:**

Accurate 3D segmentation of nasopharyngeal carcinoma (NPC) from MRI is critical for radiotherapy planning but remains challenging due to poor multi-center generalization, lack of spatial continuity, and high computational costs of global modeling. We aim to address these limitations by proposing a novel 3D framework.

**Methods:**

We propose MambaGLA-NPC, a 3D deep learning framework integrating State Space Models (SSMs) and Convolutional Neural Networks (CNNs) in a U-shaped encoder-decoder architecture. A Conv-GLSSM module jointly extracts static local features via convolutions and global context via SSMs with a 3D local scanning strategy. A gated channel-spatial attention (GCS Att) module suppresses irrelevant noise in skip connections, and an NPC-specific adapter enables parameter-efficient fine-tuning across multi-center datasets.

**Results:**

On a multi-center dataset of 1,098 NPC patients from three hospitals, our model achieves state-of-the-art performance on the internal test set (Dice: 80.47%, IoU: 67.83%, HD95: 4.09 mm). Under cross-center evaluation, it significantly outperforms prior methods (e.g., Dice of 87.13% and 76.70% on two external centers). The NPC-Adapter boosts cross-center Dice by up to 8.84% compared to no adaptation. Ablation studies confirm the contribution of each module, and the Conv-GLSSM reduces computational cost versus Transformers (21.66 GFLOPs vs. OOM for self-attention at $128^{3}$ resolution).

**Conclusion:**

MambaGLA-NPC is the first Mamba-based 3D NPC segmentation framework that effectively balances local-global feature integration with linear complexity, achieving superior accuracy and generalization across multi-center MRI data while maintaining high efficiency.

## 1 Introduction

Nasopharyngeal carcinoma (NPC) is a prevalent head and neck malignancy highly sensitive to radiotherapy (Chen et al., 2019; Tseng et al., 2020). Precise tumor target delineation is crucial for radiotherapy planning. While magnetic resonance imaging (MRI) is the primary modality for NPC diagnosis and target delineation due to its superior soft tissue contrast, manual delineation is labor-intensive, time-consuming, and highly subjective (Zhang et al., 2019). Furthermore, as shown in Figure 1, NPC tumors typically exhibit infiltrative growth patterns with blurred margins and prominent imaging heterogeneity across different stages (e.g., T2, T1C). These challenges necessitate the development of automated, high-precision segmentation methods.

**FIGURE 1:**
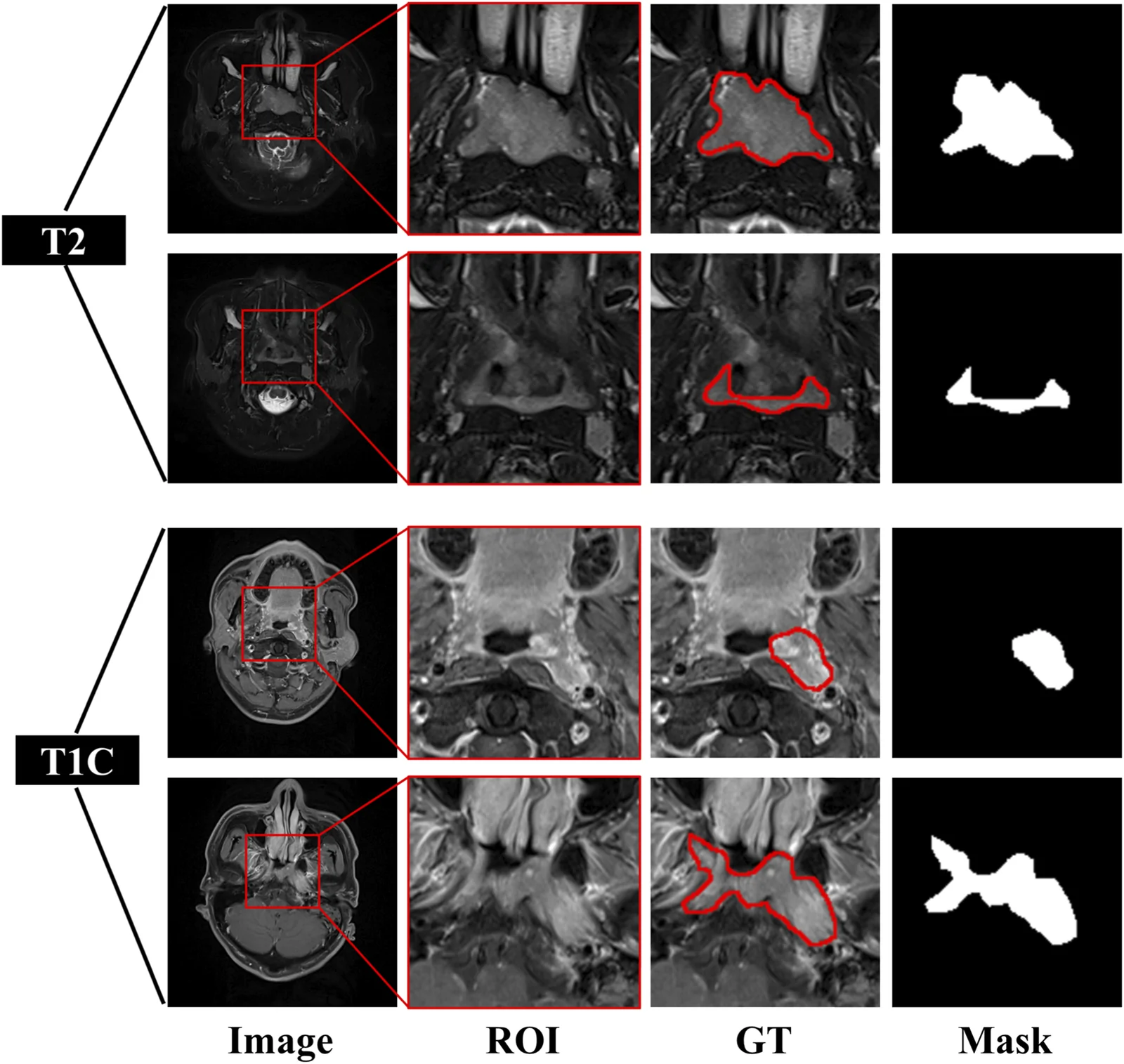
Schematic of NPC Tumor Region Outlining. The first column displays the original MRI images; the second column presents an enlarged view of the NPC region; the third column shows the tumor region boundary contours annotated by clinicians; the fourth column displays the extracted binary mask. The first two rows represent T2-weighted (T2) images, while the latter two rows depict contrast-enhanced T1-weighted (T1C) images.

Deep learning, particularly U-Net and its variants, has significantly advanced medical image segmentation (Lin et al., 2019; Khan et al., 2020; Liu et al., 2020; Ronneberger et al., 2015; Zhang et al., 2020; Hatamizadeh et al., 2022a). For NPC MRI segmentation, CNNs enhanced with attention mechanisms or dynamic kernels have shown promising results (Zhang et al., 2022; Yang et al., 2025). However, the inherent local receptive fields of CNNs struggle to model the long-range dependencies required to capture the irregular shapes and dispersed distributions of NPC tumors. Transformer-based architectures (Vaswani et al., 2017) mitigate this by modeling global context, demonstrating efficacy in recent NPC staging and segmentation tasks (Yang et al., 2024; Huang et al., 2022). However, they introduce substantial computational overhead due to the quadratic complexity of self-attention. More importantly, most existing studies are confined to 2D, single-center datasets. This reliance ignores crucial 3D tumor continuity (Cai et al., 2024) and severely limits model generalizability against domain shifts caused by diverse MRI scanners and imaging protocols (Qi et al., 2025; Zhang et al., 2025; Che et al., 2025).

Recently, state space models (SSMs), particularly Mamba (Gu and Dao, 2024), have emerged as a powerful alternative for sequence modeling, offering robust global modeling capabilities with linear computational complexity (Gu et al., 2021; 2022). This architecture has been successfully adapted for computer vision (Zhu et al., 2024; Liu et al., 2024) and medical image analysis, achieving state-of-the-art performance with significantly lower memory footprints. For instance, frameworks like SegMamba (Xing et al., 2024) and Mamba-UNet integrations (Wang Z. et al., 2024) have effectively captured long-range dependencies in 3D medical volumes. However, despite these breakthroughs, the application of Mamba-based architectures specifically tailored for 3D NPC segmentation remains unexplored.

To address these challenges, we propose MambaGLA-NPC, a novel 3D MRI-based segmentation framework for NPC. Built upon the Mamba architecture with a U-shaped design, MambaGLA-NPC seamlessly integrates local and global information. The encoder employs a novel Conv-GLSSM module with a dual-branch structure to extract multi-scale features, with a Conv branch extracting static local features and a GLSSM (Global-Local State Space Model) branch combining global modeling with 3D local scanning. This design is intended to help the network capture dynamic local features, preserve spatial continuity, and direct attention toward tumor boundary regions. The decoder subsequently aggregates semantic information using a convolutional network. The main contributions of this paper are summarized as follows:

- We introduce MambaGLA-NPC, the first Mamba-based framework designed specifically for 3D MRI semantic segmentation of NPC, effectively balancing local-global feature integration with linear computational complexity.
- We propose the Conv-GLSSM module to concurrently model static and dynamic local features alongside global context. This is further enhanced by a BiAtt module for diverse feature fusion and a gated attention aggregation module to suppress irrelevant background noise in skip connections.
- To address multi-center domain shifts, we design an NPC-Adapter, endowing the model with robust domain adaptation capabilities for diverse clinical environments.
- Extensive experiments demonstrate that MambaGLA-NPC achieves superior segmentation accuracy and better generalization on multi-center data compared to state-of-the-art methods, all while significantly reducing computational costs.

## 2 Related work

### 2.1 NPC segmentation in medical imaging

Prior to the deep learning era, NPC segmentation primarily relied on traditional machine learning methods (Zhou et al., 2006; Huang et al., 2013). However, these approaches depended heavily on sophisticated, labor-intensive manual feature engineering, limiting their scalability. Recently, deep learning has revolutionized automated NPC segmentation (Luo et al., 2022). Current mainstream approaches are dominated by Convolutional Neural Networks (CNNs) and Transformers. For instance, modified U-Net architectures (Çiçek et al., 2016; Astaraki et al., 2023) and their variants (Alom et al., 2018; Zhou et al., 2018; 2024) have been widely adopted for precise local feature extraction. In particular, multi-scale fusion CNNs, such as 3D CNNs, have demonstrated effectiveness in capturing hierarchical representations by integrating features extracted with different kernel sizes (Yang et al., 2022; Wang, 2023; Çiçek et al., 2016). To further capture broader contexts, hybrid models integrating attention mechanisms or Transformers have been proposed (Zhao et al., 2024; Zeng et al., 2024).

Despite these advancements, a fundamental dilemma remains. CNN-based models are inherently constrained by limited local receptive fields, restricting their global semantic modeling capabilities for large and irregular NPC target regions. Conversely, while the Transformer architecture (Vaswani et al., 2017) excels at capturing long-range dependencies via self-attention, it incurs a prohibitive quadratic computational complexity. This imposes extreme resource demands, particularly when processing high-resolution or 3D medical volumes, as illustrated in Figure 2.

**FIGURE 2:**
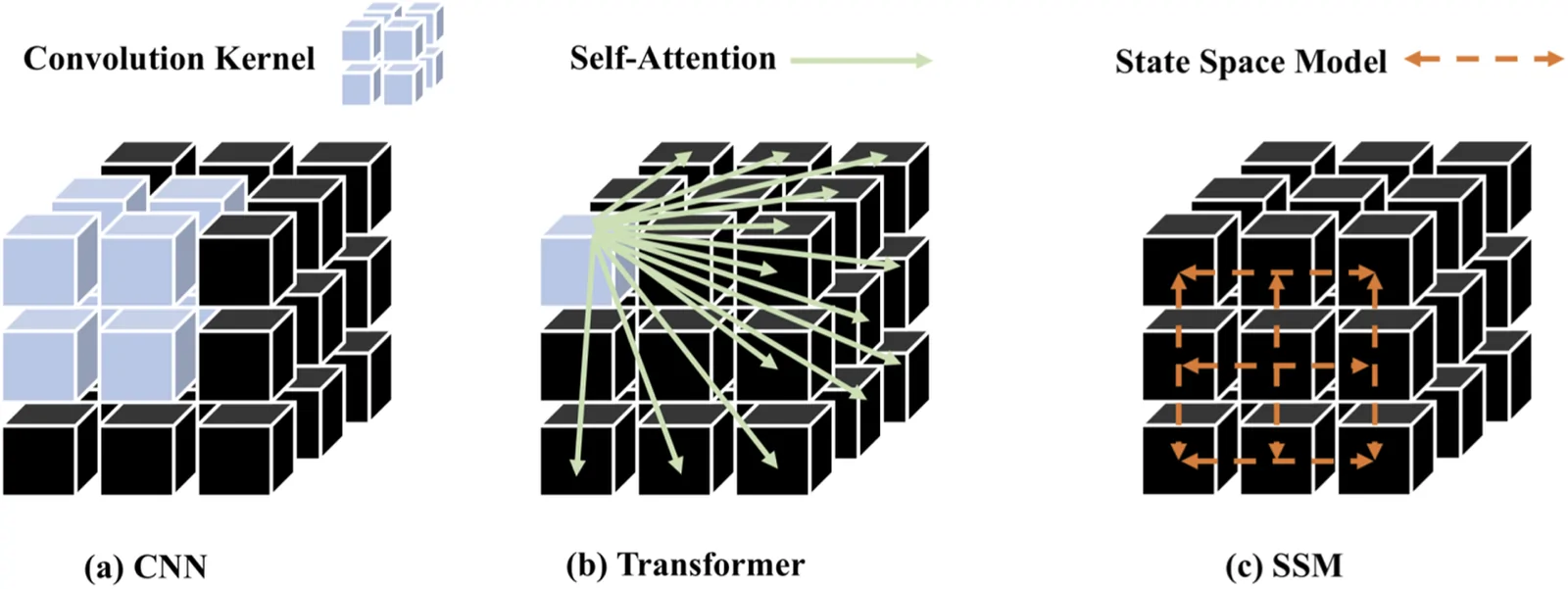
Illustration of different architectures processing 3D images. **(a)** CNN. **(b)** Transformer. **(c)** SSM.

### 2.2 Mamba architecture in medical image analysis

To overcome the computational bottleneck of Transformers, State Space Models (SSMs), particularly the Mamba architecture (Gu and Dao, 2024), have emerged as a compelling alternative. By designing a selective state space, Mamba achieves robust global receptive fields with linear computational complexity, significantly enhancing the processing efficiency for long-sequence data. The introduction of Vision Mamba (Vim) (Zhu et al., 2024) and VMamba successfully adapted SSMs for visual tasks, enabling efficient spatial feature extraction. Consequently, Mamba has been rapidly integrated into medical image analysis (Ye et al., 2024; Xing et al., 2024). Architectures like U-Mamba (Ma et al., 2024) effectively combine Mamba’s global context modeling with U-Net’s hierarchical structure, providing new technical pathways for biomedical image segmentation.

Although Mamba and its derivative architectures have shown great promise, their application to complex NPC segmentation is hindered by several critical limitations. These include weaker local fine-grained detail extraction relative to CNNs, inadequate skip connections in existing U-shaped Mamba models that fail to bridge the encoder–decoder semantic gap (thereby increasing susceptibility to irrelevant background noise in MRI scans), and the limited adaptability of standard static local feature extraction to the irregular and heterogeneous tissue structures of pathological tumors (Bansal et al., 2024; Li et al., 2025; Xing et al., 2025).

To address these limitations, we propose a hybrid 3D CNN-Mamba framework tailored for NPC. The encoder employs a dual-branch feature extraction approach, in which 3D CNNs extract static local details while a novel GLSSM branch simultaneously models dynamic local and global information.

## 3 Methods

As shown in Figure 3, our MambaGLA-NPC is built upon a U-shaped encoder-decoder architecture specifically designed for NPC segmentation tasks. It primarily consists of three components: 1) a 3D feature encoder based on multiple Conv-GLSSM layers, which simultaneously models global and local information of NPC at different scales; 2) skip connections based on gated attention to eliminate irrelevant noise; 3) an NPC-Adapter added before and after the key feature extraction module, enabling efficient model transferability across diverse datasets; 4) a decoder based on convolutional layers for predicting segmentation results. This section will further elaborate on the detailed architecture of MambaGLA-NPC.

**FIGURE 3:**
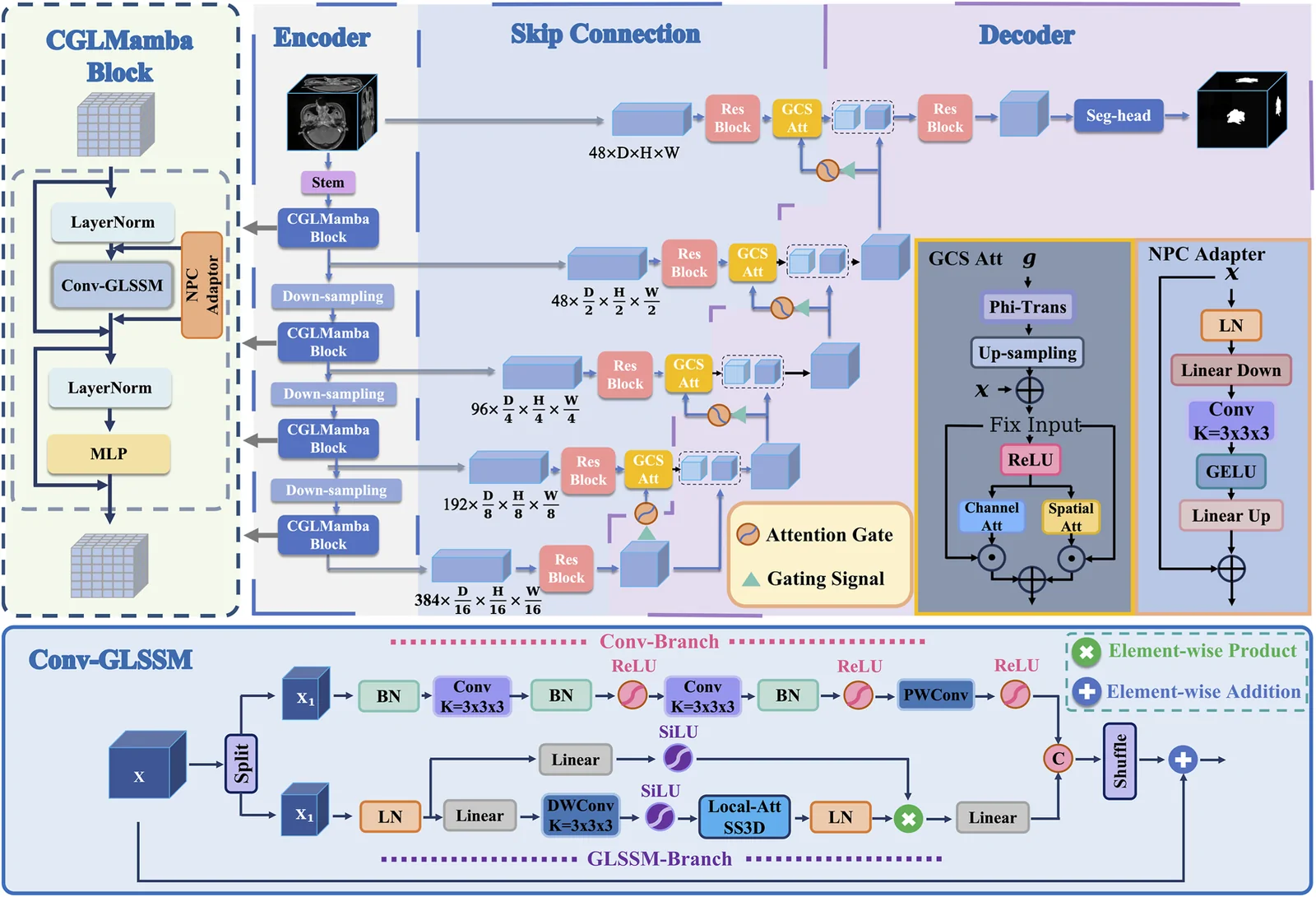
Overview of the proposed MambaGLA-NPC model. The encoder comprises a Stem layer and multiple CGLMamba blocks for multi-scale feature extraction. Within each CGLMamba block, Conv-GLSSM effectively models both local and global information in 3D MRI NPC images, while the NPC-Adapter enables efficient fine-tuning for multi-center transfer learning. Additionally, we designed the GCS Att to suppress irrelevant features in skip connections, thereby enabling high-quality decoding of compressed features.

### 3.1 CGLMamba block

Modeling global and multi-scale features is crucial for 3D NPC image segmentation. While Transformer architectures can extract global information, they exhibit significant computational overhead when processing excessively long feature sequences. To reduce sequence length, Transformer-based methods like UNETR directly downsample the 3D input resolution D × H × H to D/2 × H/2 × W/2. However, this approach limits the encoding capability of multi-scale features, which are essential for predicting segmentation results through the decoder. To overcome this limitation, we designed the CGLMamba Block, which simultaneously considers local features while achieving efficient multi-scale and global modeling, enabling the model to simultaneously model local information.

As shown in Figure 3, the encoder consists of a stem layer and multiple CGLMamba Blocks. For the Stem layer, we employ deep convolutions using a large 7×7× 7 convolution kernel, 3×3× 3 padding parameters, and a 2×2× 2 stride. Given a 3D input volume $I\in R^{C\times D\times H\times W}$ where C denotes the number of input channels, the stem layer extracts the first-scale feature $Z_{0}\in R^{48\times \frac{D}{2}\times \frac{H}{2}\times \frac{W}{2}}$ . Subsequently, $z_{0}$ is fed into each CGLMamba Block and its corresponding downsampling layer. For the $m^{th}$ CGLMamba Block, the computation can be defined as Equations 1, 2

$$z_{m}^{\prime }={\text{Adaptor }}_{m}^{2}\left(\text{Conv}-\text{GLSSM }\left({\text{Adaptor }}_{m}^{1}\left(\text{LN }\left(z_{m}\right)\right)\right)\right)+z_{m}$$ (1)

$$z_{m}^{\prime \prime }=\text{MLP }\left(\text{LN }\left(z_{m}^{\prime }\right)\right)+z_{m}^{\prime }$$ (2)

Here, LN denotes layer normalization, while MLP represents the multilayer perceptron layer, which is employed to enrich feature representations.

#### 3.1.1 Conv-GLSSM

Group convolutions employ a set of convolutions (multiple kernels per layer) to facilitate the model’s learning of various high-level and low-level features. This concept was first introduced in AlexNet (Krizhevsky et al., 2017) as an engineering compromise to deploy the model across multiple GPUs. Recent studies have demonstrated that this module can enhance model performance while reducing both parameter size and computational complexity. To enable MambaGLA-NPC to more effectively model 3D NPC images, we incorporate this classical concept into our proposed Conv-GLSSM. This serves as the core component of the encoder within MambaGLA-NPC. This approach employs channel-split to divide feature maps into two groups, then extracts local and global information from each group using Conv-branch and GLSSM-branch, respectively. This design explicitly enhances the model’s sensitivity to local spatial patterns while preserving global modeling capabilities, providing fundamental feature support for fine-grained visual tasks.

Finally, Conv-GLSSM employs channel-concatenation to restore channel dimension size, while channel-shuffle rearranges feature maps along channel dimension to prevent information loss between channels caused by grouped convolution operations (Zhang et al., 2018; Ma et al., 2018). Following the settings of classical CNNs and VIT, we set the activation functions of Conv-Branch and GLSSM-Branch to ReLU (Agarap, 2018; Elfwing et al., 2018).

We formally define the feature map modeling process for Conv-GLSSM. First, the module’s input $x\in R^{D\times H\times W\times C}$ undergoes channel splitting $\emptyset_{\mathrm{channel-split}}$ to yield $x_{1}$ , $x_{2}$ , and then there is Equation 3:

$$\emptyset_{\mathrm{channel-split}}\left(x\right)\to x_{i}\;\;\;\;x_{i=1,2}\in R^{D\times H\times W\times \frac{C}{2}}$$ (3)

Next, $x_{1}$ , $x_{2}$ are input into Conv-Branch $\emptyset_{\mathrm{Conv-Branch}}$ and GLSSM-Branch $\emptyset_{\mathrm{GLSSM-Branch}}$ respectively to obtain $\tilde{x_{1}}$ and $\tilde{x_{2}}$ . This process can be represented as Equation 4:

$$\tilde{x_{1}}{=\emptyset }_{\mathrm{Conv-Branch}}\left(x1\right)\;\;\;\;\tilde{x_{2}}{=\emptyset }_{\mathrm{GLSSM-Branch}}\left(x2\right)$$ (4)

Finally, we perform channel concatenation $\emptyset_{\mathrm{channel-concatenation}}$ and channel shuffle $\emptyset_{\mathrm{channel-shuffle}}$ on the outputs of the two branches, then add them to the initial input x to obtain the final output feature map y (Equation 5):

$$y=x⊕\emptyset_{channel-shuffle}\left(\emptyset_{channel-concatenation}\left({\tilde{x}}_{1},{\tilde{x}}_{2}\right)\right)$$ (5)

##### 3.1.1.1 Conv branch

In Visual SSMs, the modeling capability of global long-range dependencies significantly enhances the model’s understanding of overall image structure. However, its ability to capture local fine-grained features—such as the complex, blurred boundaries of NPC—remains relatively limited. To address this shortcoming, we introduce the Conv-Branch module. Conv-Branch applies successive 3D convolution layers with ReLU activation to extract local features, and uses a Pointwise Convolution (PWConv, i.e., 1×1×1 convolution) for cross-channel semantic fusion. For the input $x_{1}$ to Conv-Branch, we employ a permutation operation to rearrange the original feature map to match the convolutional operation. Its modeling process can be defined as follows Equations 6–11:

$$permute\left(x_{1}\right)\to \bar{x_{1}}\in R^{\frac{C}{2}\times D\times H\times W}$$ (6)

$$x_{1}^{\prime }={BatchNorm}_{1}\left(\bar{x_{1}}\right)$$ (7)

$$x_{1}^{\prime \prime }=\left(ReLU{BatchNorm}_{2}\left({Conv}_{3\times 3\times 3}\left(x_{1}^{\prime }\right)\right)\right)$$ (8)

$$x_{1}^{\prime \prime \prime }=\left({ReLUBatchNorm}_{2}\left({Conv}_{3\times 3\times 3}\left(x_{1}^{\prime \prime }\right)\right)\right)$$ (9)

$$\hat{x_{1}}=ReLU\left(PWConv\left(x_{1}^{\prime \prime \prime }\right)\right)$$ (10)

$$permute\left(\hat{x_{1}}\right)\to \tilde{x_{1}}\in R^{D\times H\times W\times \frac{C}{2}}$$ (11)

##### 3.1.1.2 LocalAtt SS3D

To model the local spatial continuity of 3D medical images, our method divides the image into multiple distinct 3D local windows. This ensures a closer arrangement of relevant 3D local tokens, thereby enhancing the capture of 3D local dependencies. Our approach is compared with prior methods that fail to preserve local spatial continuity (as shown in Figure 4).

**FIGURE 4:**
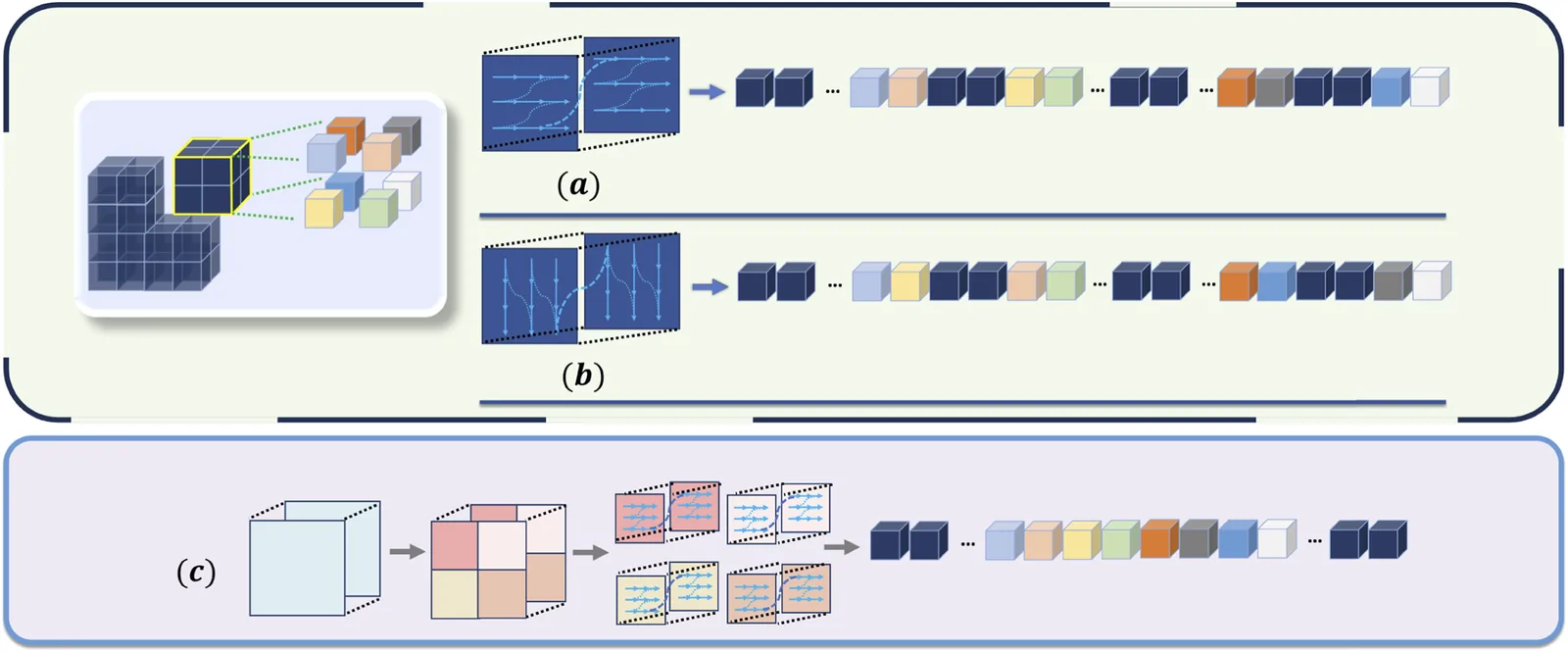
Schematic of scanning methods. **(a,b)** Previous methods Vim (Zhu et al., 2024) and VMamba (Liu et al., 2024) scan along entire row or column axes, resulting in significant distance when capturing dependencies between consecutive pixels within the same semantic region. **(c)** We propose a novel scanning method that divides tokens into independent windows (each window sized 8×8×8 ) and scans within each window. This approach enhances the ability to capture local dependencies.

While our approach excels at effectively capturing local dependencies within each region, we also recognize the importance of global context. To this end, we construct the base block by integrating selective scanning mechanisms across four directions: the original line-by-line scan and the proposed 3D local scan, along with their reversed counterparts to enable tail-to-head scanning (the reverse direction is adopted in Vim and VMamba to better model non-causal image tokens). This multidimensional approach ensures comprehensive analysis within each selective scanning block, achieving a balance between local detail and global perspective.

As shown in Figure 5a, our module processes each input image feature through four independent selective scan branches. These branches capture relevant information separately, which is subsequently merged into a unified feature output. To enhance the integration of diverse features and eliminate redundant information, we introduce the Bidirectional Attention Mechanism (BiAtt) module prior to merging.

**FIGURE 5:**
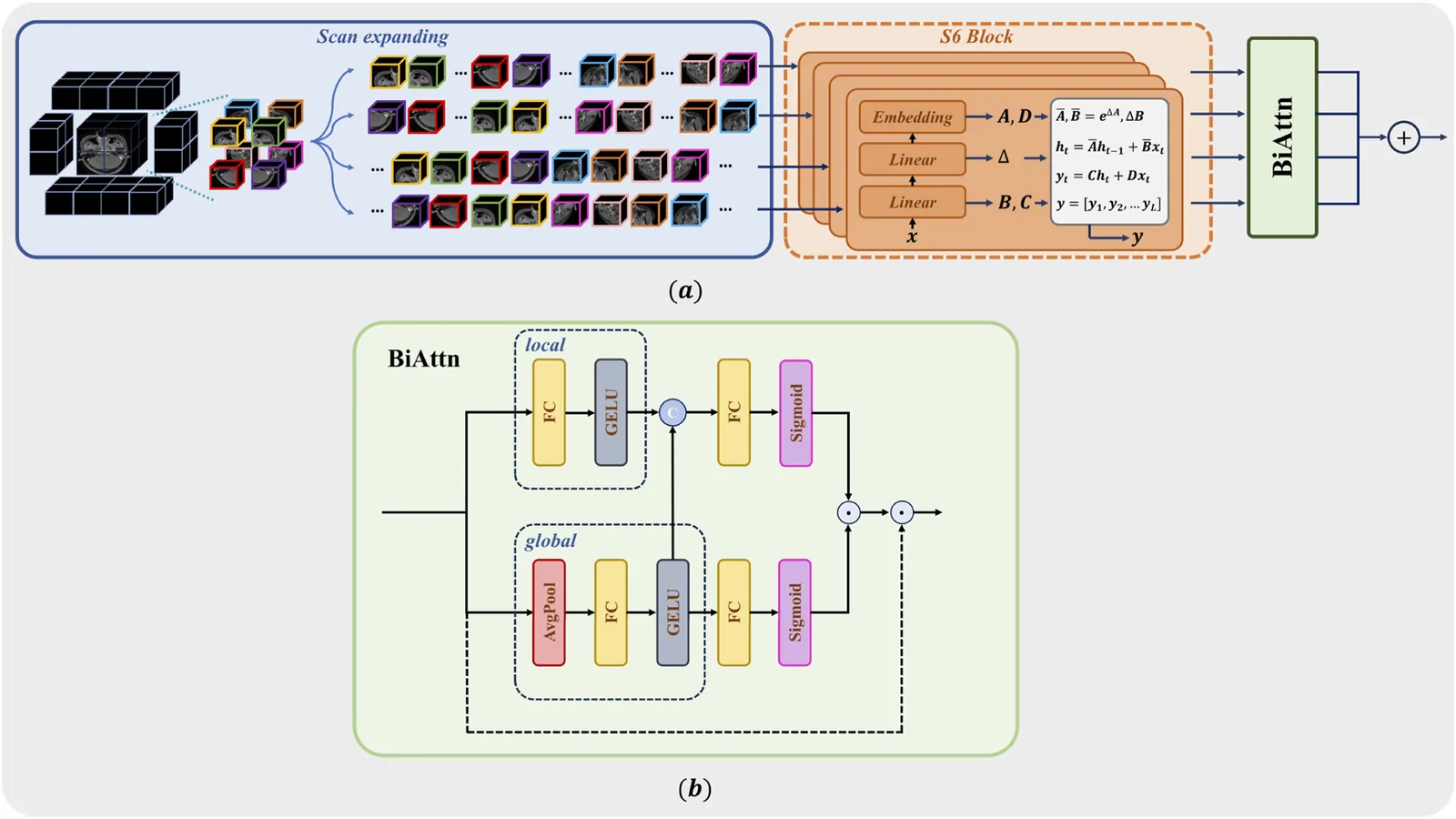
**(a)** Visualization of the internal modeling process of LocalAtt SS3D. **(b)** Schematic diagram of the proposed bidirectional attention mechanism module.

As shown in Figure 5b, this module processes channels and tokens within each branch feature through adaptive weighting, comprising two core components: the channel attention branch and the spatial attention branch. The channel attention branch generates a global representation by averaging the input features across the spatial dimension, then applies a linear transformation to determine the channel weights. Conversely, the spatial attention mechanism evaluates token-level importance by combining each token’s feature with the global representation, enabling fine-grained, importance-based feature extraction.

### 3.2 GCS att

To capture sufficiently large receptive fields and thereby obtain semantic contextual information, feature maps in standard CNN architectures are progressively downsampled. This allows coarse spatial grid-level features to model the spatial relationships and mutual positions of biological tissues at global scales. However, for small objects with high shape variability, reducing false positive predictions remains challenging. To enhance accuracy, current segmentation frameworks rely on additional pre-processing object localization models, simplifying the task into separate localization and subsequent segmentation steps. Here, we achieve the same goal by integrating Gate-Controlled Spatial Channel Attention (GCS Att) into a standard CNN model. Unlike localization models in multi-stage convolutional neural networks, GCS Att progressively suppresses feature responses from irrelevant background regions without requiring region-of-interest (ROI) cropping between networks.

Specifically, information extracted from coarse scales is used for gating to eliminate irrelevant noise and responses in skip connections. This operation is performed prior to the connection operation to merge only relevant activations. Additionally, the gating mechanism filters neuronal activations during both forward and backward propagation. Gradients originating from background regions are weighted down during backpropagation. This ensures model parameters in shallower layers are updated primarily based on spatially relevant regions related to the given task. Feature maps obtained through gating are fed into spatial-channel attention. Both spatial and channel attention apply max-pooling and average-pooling to capture image texture and contextual information, significantly enhancing the model’s perception of key structures. The processing steps for the input feature map $X\in R^{B\times C\times D\times H\times W}$ and the gated input $G\in R^{B\times C_{g}\times D_{g}\times H_{g}\times W_{g}}$ are as follows Equations 12–17:

$$G^{\prime }=\varphi \left(G\right)$$ (12)

$$G_{\mathrm{align}}=u\left(G^{\prime },\;size=\left(D,H,W\right)\right)$$ (13)

$$F=ReLU\left(X⊕G_{\mathrm{align}}\right)$$ (14)

$$C_{\max}=\underset{\left(d,h,w\right)}{\text{MaxPool}}\left(F\right),C_{\text{avg}}=\underset{\left(d,h,w\right)}{\text{AvgPool}}\left(F\right),A_{c}=\sigma \left(\text{MLP}\left(C_{\max}\right)+\text{MLP}\left(C_{\text{avg}}\right)\right)$$ (15)

$$S_{\max}=\max_{c\in \left[1,C\right]}\left(F\right),S_{\text{avg}}=\frac{1}{C}\overset{C}{\underset{c=1}{\sum }}\left(F\right),\;A_{s}=\text{Conv }\left({\text{Cat }}_{\dim=1}\left(S_{\max},S_{\text{avg}}\right)\right)$$ (16)

$$Y=A_{c}⊙F+A_{s}⊙F$$ (17)

### 3.3 Adapter in MambaGLA-NPC (NPC adapter)

In the field of medical image analysis, particularly for 3D segmentation tasks targeting specific pathologies such as NPC, acquiring large-scale, high-quality annotated data remains highly challenging. When pre-trained models encounter data distribution shifts from new institutions, new scanning protocols, or different patient cohorts, traditional full fine-tuning often leads to two critical issues: Over-adjusting pre-trained weights may destroy the model’s learned general anatomical feature representations from source data, while limited domain-specific data struggles to fully optimize all parameters of high-capacity models. To address these challenges, we introduce the Parameter-Efficient Fine-Tuning (PEFT) strategy (Xin et al., 2026), specifically employing an Adapter module to achieve efficient adaptation. The core idea is to freeze all parameters of the pre-trained backbone to preserve general knowledge, while inserting lightweight learnable Adapter layers (referred to as Pre-Mamba Adapter and Post-Mamba Adapter) before and after the core feature extraction module Conv-GLSSM. These two newly added modules are fine-tuned on the target data. We refer to this fine-tuning strategy as the NPC Adapter. This approach significantly reduces trainable parameters while enhancing the model’s adaptability to new data, effectively suppressing overfitting and accelerating convergence.

Our NPC Adapter features a simple architecture where each Adapter serves distinct functions. The Pre-Mamba Adapter purifies domain shifts introduced by variations in scanning equipment and protocols in the raw data. The Post-Mamba Adapter transforms the general features extracted by Conv-GLSSM into task-oriented decision features. This cascaded adaptation pipeline comprehensively addresses shifts from data to domain. For input $X\in R^{B\times C\times D\times H\times W}$ , processing through the Adapter yields A(X) (Equation 18):

$$A\left(X\right)=X+L_{up}\left(GELU\left(Conv\left(L_{\mathrm{down}}\left(IN\left(X\right)\right)\right)\right)\right)$$ (18)

Among these, IN denotes the InstanceNorm layer, while $L_{up}$ and $L_{\mathrm{down}}$ represent the upsampling fully connected layer and the downsampling fully connected layer, respectively.

### 3.4 Decoder

MambaGLA-NPC employs a U-shaped network architecture, where feature representations extracted by the encoder at each resolution are passed to the decoder via skip connections. At each stage i(iϵ0,1,2,3) in the encoder and the bottleneck (i = 4), the output feature representation is reshaped to $\frac{D}{2^{i}}\times \frac{H}{2^{i}}\times \frac{W}{2^{i}}$ and fed into a residual block composed of two 3×3× 3 convolutional layers, normalized by instance normalization layers. Subsequently, a deconvolution layer doubles the resolution of the feature map, and the input is concatenated with the output from the previous stage. The concatenated features are then fed into another residual block as described previously. The final segmentation is computed via a 1×1× 1 convolution layer followed by a sigmoid activation function.

### 3.5 Loss function

To address the core challenge in 3D NPC segmentation, particularly the extreme class imbalance between minute tumor volumes and vast background tissue, we employ a hybrid loss function to optimize the model. This hybrid loss function integrates Focal Loss (Lin et al., 2017) and Dice Loss (Milletari et al., 2016), designed to simultaneously tackle the dual challenges of foreground-background voxel imbalance and precise boundary delineation.

Severe class imbalance (where tumor voxels constitute only a tiny fraction of the entire 3D volume) causes model training to be dominated by the background class, making standard cross-entropy loss ineffective under these conditions. Therefore, we adopt Focal Loss, which introduces a modulation factor to reduce the loss weight of correctly classified negative samples, enabling the model to focus on difficult-to-classify samples (Equation 19):

$$L_{\text{focal}}=-\frac{1}{N}\overset{N}{\underset{i=1}{\sum }}\alpha {\left(1-p_{i}\right)}^{\gamma }\log\left(p_{i}\right)$$ (19)

Here, $p_{i}$ is the model’s predicted probability for the true class. The focus parameter γ≥0 reduces the relative loss for easily classified samples, forcing the model to learn from more difficult-to-classify samples. The balance factor α∈[0,1] is used to address class imbalance. Following (Lin et al., 2017), we set γ=2.0 and α=0.25 .

Focal Loss effectively addresses class imbalance, while Dice Loss directly optimizes the overlapping region between predicted and ground truth labels—a goal highly relevant to core segmentation evaluation metrics. This makes it particularly suitable for medical image segmentation tasks, where the geometric integrity of objects is critical (Equation 20):

$$L_{dice\;}=1-\frac{2\overset{N}{\underset{i=1}{\sum }}p_{i}g_{i}+\epsilon }{\overset{N}{\underset{i=1}{\sum }}p_{i}+\overset{N}{\underset{i=1}{\sum }}g_{i}+\epsilon }$$ (20)

In this equation, $p_{i}\in \left[0,1\right]$ and $g_{i}\in 0,1$ represent the predicted probability and true label of voxel i, respectively. The smoothing factor $\epsilon =1\times 10^{-5}$ is employed to prevent division-by-zero errors and ensure numerical stability.

The final loss function comprises Dice Loss and Focal Loss, which are used to supervise model training (Equation 21):

$$Loss=Loss_{\mathrm{Dice}}+Loss_{\mathrm{Focal}}$$ (21)

## 4 Experiment

### 4.1 Experiment setups

#### 4.1.1 Datasets

We conducted experiments on MRI from 1,098 patients with pathologically and clinically confirmed locally advanced NPC, collected between January 2015 and April 2020 at three hospitals: Guangxi Medical University Cancer Hospital (TW-Center, n = 434), Wuzhou Red Cross Hospital (TH-Center, n = 566), and Guangxi Medical University Second Affiliated Hospital (FI-Center, n = 98). Patients were included if they had a measurable primary tumor ( > 5 mm) with complete clinical data and pretreatment MR images of sufficient quality, had not received antineoplastic therapy before MR examination, and had no pre-existing or concurrent other primary malignant tumors. Briefly, the cohort comprised 839 male (76.4%) and 259 female (23.6%) patients with a median age of 47 years (IQR: 38–54). The T-stage distribution was T1: 3.2%, T2: 23.6%, T3: 32.2%, T4: 41.0%; the N-stage distribution was N0: 1.3%, N1: 14.9%, N2: 53.0%, N3: 30.8%; and 95.2% of tumors were WHO type III. These MR images included T2 and T1C sequences which were annotated independently by two radiologists with 8 years of experience in head and neck MRI diagnosis. Irrelevant regions such as air and bone were excluded through manual adjustments. All segmentation results were reviewed and confirmed by a senior radiologist with 25 years of head and neck MRI diagnostic experience, and discrepancies were resolved through consensus discussion. Inter-reader agreement between Reader A and Reader B was assessed using the intra-class correlation coefficient (ICC). Fifty patients were randomly selected from the training cohort, and both readers independently segmented the VOIs. Features with ICC values exceeding 0.75 were considered to have good reproducibility. All radiologists were blinded to the patients’ clinical and pathological information. Imaging parameters at each hospital are as follows:

##### 4.1.1.1 Guangxi Medical University cancer hospital

Patients were examined using a 3.0T MRI scanner (GE Healthcare Discovery MR750, USA). T2WI and CET1WI were obtained. The parameters were listed as follows: T2WI (TR = 6,760 m, TE = 91 m), CET1WI (TR = 957 m, TE = 19 m), matrix size = 256×256 , FOV = 240 mm × 240 mm, slice thickness = 5 mm, flip angle = 90 ° , and slice spacing = 1.0 mm. The contrast agent administered was Gd-DTPA (Magnevist meglumine, Bayer Healthcare Pharmaceuticals, Berlin, Germany), which was given at a dose of 0.1 mmol/kg body weight (flow rate: 2.0 mL/s).

##### 4.1.1.2 Wuzhou Red Cross Hospital

Patients were examined using a 3.0T MRI scanner (Magnetom Skyra, Siemens Healthcare, Germany). T2WI and CET1WI were obtained. The parameters were listed as follows: T2WI (TR = 3,500 m, TE = 95 m), CET1WI (TR = 600 m, TE = 11 m), matrix size = 240×320 , FOV = 230 mm × 230 mm, slice thickness = 5 mm, flip angle = 90 ° , and slice spacing = 0.5 mm. The contrast agent administered was Gd-DTPA (Magnevist meglumine, Bayer Healthcare Pharmaceuticals), which was given at a dose of 0.1 mmol/kg body weight (flow rate: 2.0 mL/s).

##### 4.1.1.3 The Second Affiliated Hospital of Guangxi Medical University

Patients were examined using a 3.0T MRI scanner (GE Healthcare Discovery MR750). T2WI and CET1WI were obtained. The parameters were listed as follows: T2WI (TR = 7,061 m, TE = 68 m), CET1WI (TR = 643 m, TE = 11 m), matrix size = 288×192 , FOV = 220 mm × 220 mm, slice thickness = 4 mm, flip angle = 111 ° , and slice spacing = 0.4 mm. The contrast agent administered was Gd-DTPA (Magnevist meglumine, Bayer Healthcare Pharmaceuticals), which was given at a dose of 0.1 mmol/kg body weight (flow rate: 2.0 mL/s).

In our experimental design, we divided the patients from each hospital into training and testing sets at a 9:1 ratio. The specific quantities are shown in Table 1. To conduct both internal and external testing simultaneously, we adopted a center-based partitioning strategy: the model was trained only on the training dataset from a randomly selected center, followed by internal testing on that center’s test dataset. External testing was then performed on the previously unseen data from the other two centers. We randomly selected TH-Center (n = 509) as the training data, TH-Center (n = 57) as the internal test data, and FI-Center and TW-Center (n = 54) as the external test data. TH-Center was selected as the source domain because it provided the largest training set among the three centers, which helps ensure sufficient data for model training. This selection was made prior to any model training or comparison, to avoid selection bias. The flowchart is shown in Figure 6.

**TABLE 1:** Specific patient numbers of training and test datasets at each center.

Center	Training set	Testing set
FI-Center	88	10
TW-Center	390	44
TH-Center	509	57

**FIGURE 6:**
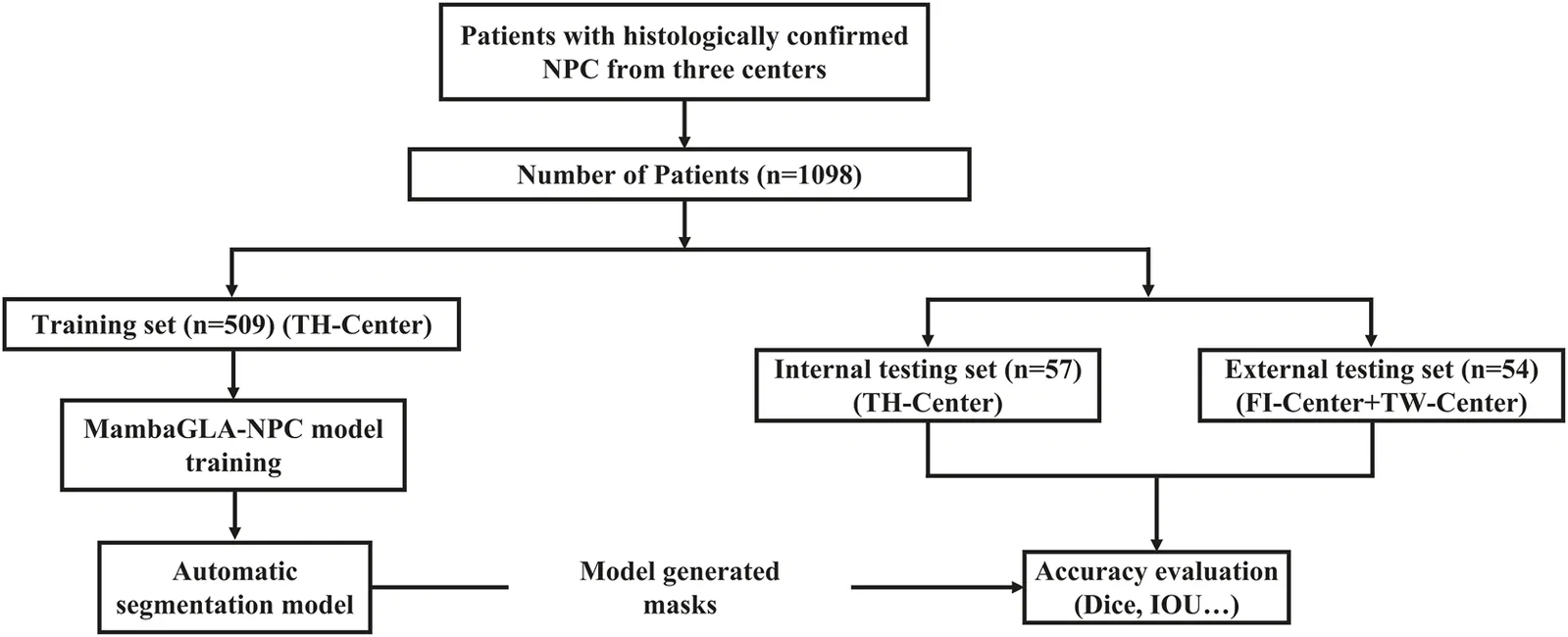
Flowchart of this study.

#### 4.1.2 Metrics

To evaluate the accuracy of our segmentation method, we performed multi-metric calculations comparing the masks generated by MambaGLA-NPC segmentation with the ground truth. Segmentation metrics are as follows: $Dice=\frac{TP*2}{TP*2+FP+FN+1e-7}$ , $IoU=\frac{TP}{TP+FN+FP+1e-7}$ , $Recall=\frac{TP}{TP+FN+1e-7}$ , $HD95=\max\left(h\left(A,B\right),h\left(B,A\right)\right)$ (Taha and Hanbury, 2015; Gao et al., 2025), TP, TN, FP, and FN denote true positive, true negative, false positive, and false negative, respectively. h (A,B) represents the 95th percentile Hausdorff distance between the predicted mask A and the ground truth mask B. The Intersection over Union (IoU) is the ratio of the intersection area between the predicted mask and the ground truth mask to their combined area, indicating the degree of overlap. Higher recall indicates more detected objects. Dice coefficient and IoU are widely applied in medical image segmentation tasks, where higher values represent greater overlap between predicted and ground truth values. The 95% Hausdorff distance (HD95) evaluates morphological accuracy by measuring the maximum mismatch between the boundaries of two point sets. Lower values indicate better matching between predicted and ground truth outlines. All metrics were computed on a per-patient basis, and the reported values represent the mean across all test patients.

#### 4.1.3 Implementation

Prior to model training, multi-modal MRI preprocessing was executed through a standardized pipeline. N4 bias field correction was first applied to eliminate low-frequency intensity inhomogeneities across the scans. For each patient, the T1C volume was rigidly registered to the corresponding T2 reference using elastix, after which all volumes were resampled to an isotropic voxel spacing of $1\times 1\times 1\;mm^{3}$ using B-spline interpolation for MR sequences and nearest-neighbor interpolation for ground-truth masks. Intensity values were then standardized via z-score normalization based on foreground voxel distributions. During training, random sub-volumes of size 128×128×128 were cropped from the volumetric data, with data augmentation performed via random axial flipping and intensity scaling within a ±10% range.

We employed the AdamW optimizer (Loshchilov and Hutter, 2019) with a base learning rate set to 0.0001 and a weight decay of $1\times 10^{-5}$ . This algorithm combines the strengths of Adagrad and RMSprop, automatically adjusting the learning rate during training to accelerate convergence. Training employs an auto-mixed precision strategy, with a batch size of 2, determining the number of training epochs based on the stability of loss value changes, typically ranging between 50 and 100 epochs. A fixed random seed (42) was used for all experiments to ensure reproducibility. The model is implemented using PyTorch and trained on an RTX A5000 24GB GPU. The final layer applies a Sigmoid activation to produce probability maps, which are then binarized using a threshold of 0.5 to obtain the segmentation masks for evaluation.

For fair comparison, all baseline models were trained from scratch on the same TH-Center training set using the same data splits, optimizer, learning rate, and training iterations as our proposed model. No pre-trained weights were used for any model. Cross-center evaluations as shown in Table 2 were performed by directly applying the TH-Center-trained models to the FI-Center and TW-Center test sets without any domain adaptation, except for our model’s NPC-Adapter experiments which are reported separately in Section 4.4.

**TABLE 2:** Quantitative comparison of different segmentation models across centers. The default scanning strategy used in the full model is Local Scanning + Vertical Scanning. The best result is highlighted in bold, and the second-best result is underlined.

Model	FI-center				TW-center			
​	Dice(%)	IOU(%)	Recall(%)	HD95(mm)	Dice(%)	IOU(%)	Recall(%)	HD95(mm)
3D UNet (Çiçek et al., 2016)	55.00	41.63	49.89	19.30	61.69	48.00	61.48	10.20
SAM-Med3D (Wang H. et al., 2024)	67.36	51.79	61.70	10.70	69.61	53.96	67.87	6.20
UNETR (Hatamizadeh et al., 2022b)	62.62	46.49	59.73	14.85	71.72	56.35	72.36	7.67
nn-Former (Zhou et al., 2021)	66.49	50.89	66.49	13.10	72.41	57.53	73.99	7.20
DenseV-Net (Gibson et al., 2018)	72.77	58.06	70.98	7.73	72.77	58.06	70.98	7.73
PMFS-Net (Zhong et al., 2025)	61.03	46.01	64.49	13.51	80.02	67.76	81.16	4.27
Swin UNETR (Hatamizadeh et al., 2022a)	72.19	57.02	76.18	9.11	80.69	68.07	81.34	4.08
3D UXNet (Lee et al., 2022)	71.23	56.39	67.48	10.12	79.78	66.84	80.11	4.29
SegMamba (Xing et al., 2024)	76.12	62.19	77.46	6.71	81.59	69.23	81.90	3.94
MedNeXt (Roy et al., 2023)	76.07	62.13	75.36	7.40	86.56	76.61	85.94	2.59
**MambaGLA-NPC (Ours)**	**76.70**	**63.04**	**78.04**	**6.60**	**87.13**	**77.46**	**88.21**	**2.48**

### 4.2 Overall performance

This section comprehensively evaluates the segmentation performance of the proposed model using three NPC datasets from different centers (FI-Center, TW-Center, and TH-Center). Multiple evaluation metrics are employed for comparison, including Dice similarity coefficient, IOU, recall rate, and HD95. Detailed results are presented in Tables 2, 3. This evaluation focuses on comparing the segmentation accuracy and lesion detection capabilities of the proposed model against existing state-of-the-art methods.

**TABLE 3:** Quantitative comparison of different segmentation models at a single center. The default scanning strategy used in the full model is Local Scanning + Vertical Scanning. The best result is highlighted in bold, and the second-best result is underlined.

Model	Dice (%)	IOU(%)	Recall (%)	HD95 (mm)
3D UNet (Çiçek et al., 2016)	61.35	47.20	60.24	11.12
SAM-Med3D (Wang H. et al., 2024)	69.71	54.17	67.66	6.84
UNETR (Hatamizadeh et al., 2022b)	71.54	56.24	71.66	8.12
nn-Former (Zhou et al., 2021)	72.46	57.40	73.58	7.86
DenseV-Net (Gibson et al., 2018)	72.77	58.09	70.98	7.73
PMFS-Net (Zhong et al., 2025)	73.70	59.47	74.60	7.21
Swin UNETR (Hatamizadeh et al., 2022a)	76.98	63.19	78.21	6.08
3D UXNet (Lee et al., 2022)	75.97	61.75	76.97	6.14
SegMamba (Xing et al., 2024)	78.35	64.98	79.05	5.51
MedNeXt (Roy et al., 2023)	78.83	65.52	78.74	4.75
**MambaGLA-NPC (Ours)**	**80.47**	**67.83**	**82.21**	**4.09**

#### 4.2.1 Quantitative analysis (single-center evaluation)

As shown in Table 3, when evaluated on a patient-wise basis across the TH-Center internal test cohort ( n=57 patients), our proposed MambaGLA-NPC achieved a mean Dice score of 80.47% (95% CI: [79.35%, 81.59%]) and an IoU of 67.83%, substantially outperforming traditional CNN-based architectures such as 3D UNet (Dice: 61.35%, IoU: 47.20%) and Transformer variants like Swin UNETR (Dice: 76.98%, IoU: 63.19%). Furthermore, our model achieved a Recall of 82.21% and a superior HD95 of 4.09 mm (95% CI: [3.78, 4.41] mm). Per-patient paired Wilcoxon signed-rank tests confirmed that MambaGLA-NPC statistically significantly outperformed the second-best baseline MedNeXt (Dice: 78.83%, 95% CI: [77.68%, 79.98%]; HD95: 4.75 mm, 95% CI: [4.38, 5.12] mm; Bonferroni-adjusted p<0.001 ). These results demonstrate that the proposed framework generates segmentation masks with tighter boundary alignment and higher morphological fidelity, effectively capturing the complex infiltrative structures of NPC lesions.

#### 4.2.2 Quantitative analysis (cross-center evaluation)

Table 2 demonstrates the performance of our segmentation model when trained on the TH-Center dataset (n=509) and directly applied to two additional external medical centers for zero-shot testing (FI-Center with n=10 patients and TW-Center with n=44 patients, totaling n=54 external test patients) (Taha and Hanbury, 2015). Our results indicate that despite cross-scanner domain gaps, MambaGLA-NPC achieves favorable cross-center performance across both cohorts. Specifically, on the FI-Center test set, our model achieved a mean Dice score of 76.70% (95% CI: [73.85%, 79.55%]), an IoU of 63.04% (95% CI: [59.72%, 66.36%]), and an HD95 of 6.60 mm (95% CI: [5.62, 7.58] mm). Compared to PMFS-Net, our model achieved a 6.23% higher Dice coefficient, a 7.68% higher IoU, and a 4.44 mm reduction in HD95. This substantial performance gap indicates that while PMFS-Net surpasses our model in Recall (84.49% vs. 78.04%), this comes at the cost of significantly reduced overall segmentation quality and boundary accuracy. Furthermore, at TW-Center, our model achieves favorable performance across all four metrics, yielding a Dice score of 87.13% (95% CI: [85.68%, 88.58%]), an IoU of 77.46% (95% CI: [75.62%, 79.30%]), a Recall of 88.21%, and an HD95 of 2.48 mm (95% CI: [2.14, 2.82] mm). Per-patient paired Wilcoxon signed-rank tests confirmed that the overall performance advantages of MambaGLA-NPC over the respective second-best baselines remained statistically significant across both external cohorts (Bonferroni-adjusted p=0.012 on FI-Center vs. SegMamba and p<0.001 on TW-Center vs. MedNeXt). These results firmly demonstrate the robust generalizability and architectural superiority of our hybrid local-global modeling under multi-center distribution shifts.

#### 4.2.3 Qualitative analysis

To more clearly demonstrate the advantages of this method, we present a qualitative comparison of different approaches in Figure 7. Additionally, to better observe segmentation details, we represent the segmentation results of different models with red regions and the ground truth with purple regions. The visual results highlight the superiority of our approach over existing solutions. When applied to challenging medical images where background and targets are highly similar, most models produce incorrect segmentation, particularly UNETR and 3D UNet, generating low-quality segmentation results. In contrast, our model focuses attention more precisely on the target region, yielding higher-quality masks. Furthermore, compared to the strong baselines SegMamba and MedNeXt, our model produces more complete segmentation masks with reduced misclassification rates while maintaining higher boundary similarity.

**FIGURE 7:**
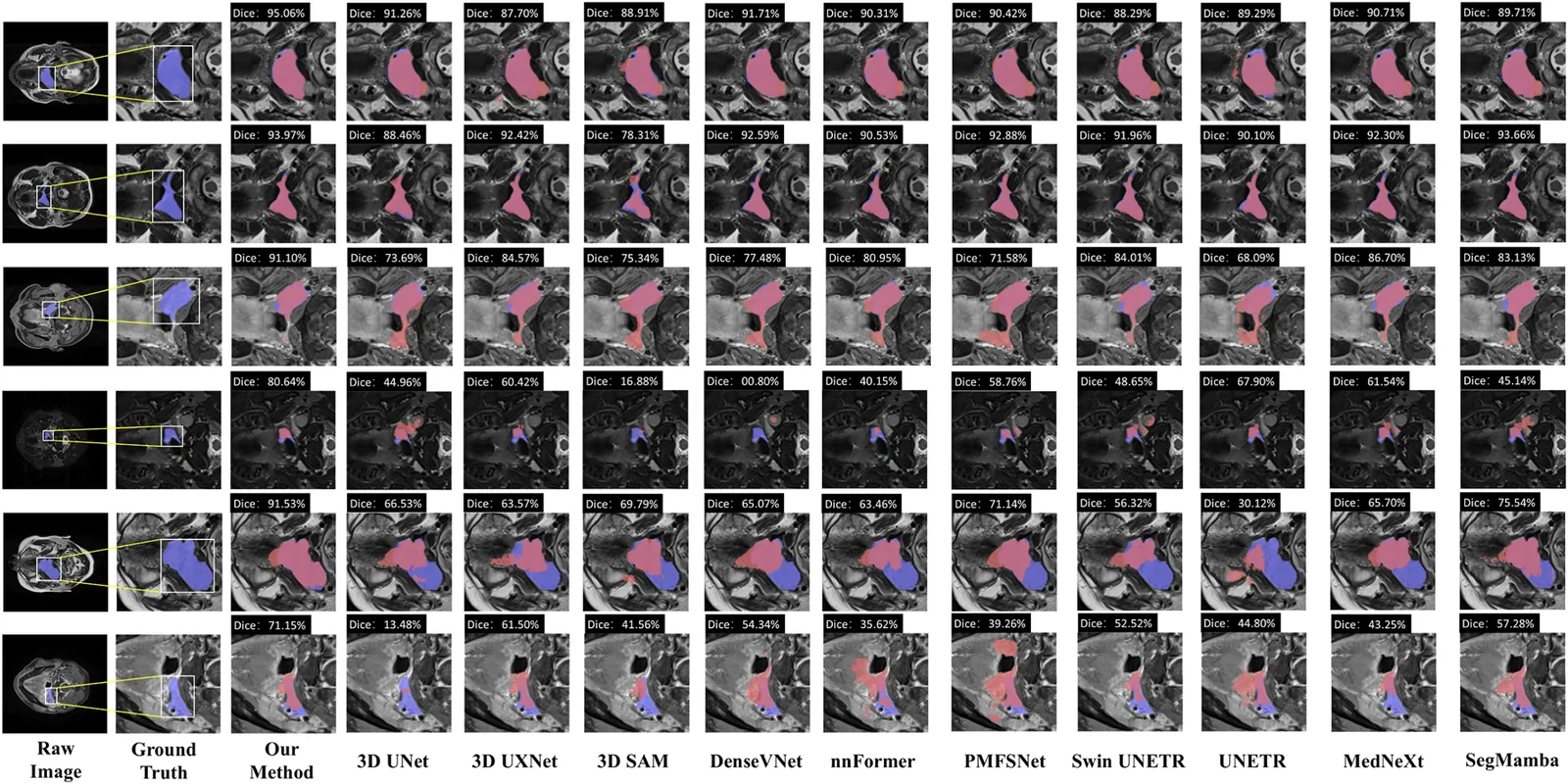
Qualitative comparison of different segmentation models, where the numbers in the upper left corner of each image represent the Dice score relative to the ground truth.

To further illustrate the model’s capability in preserving 3D spatial continuity and capturing intricate volumetric geometries, we provide 3D surface renderings of representative test cases across varying tumor volumes in Figure 8. Benefiting from the Conv-GLSSM module’s complementary local-global scanning, MambaGLA-NPC accurately reconstructs the irregular morphological contours of NPC. Specifically, for challenging micro-lesions (e.g., $0.6\;{\text{cm}}^{3}$ ) and highly infiltrative macro-tumors (e.g., $19.4\;{\text{cm}}^{3}$ and $21.9\;{\text{cm}}^{3}$ ), our model yields high geometric fidelity and smooth 3D boundary delineation with minimal over- or under-segmentation, closely aligning with the clinician-delineated ground truth.

**FIGURE 8:**
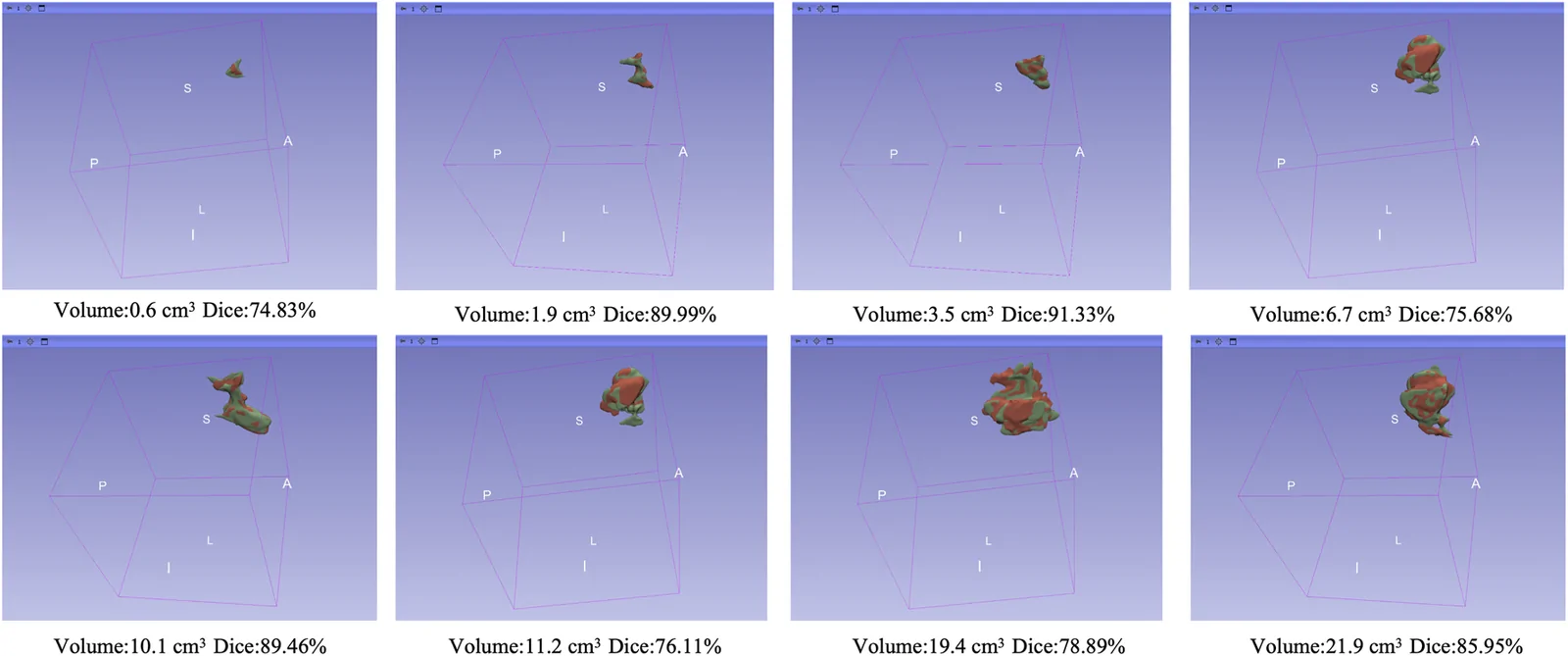
3D volumetric surface renderings of representative NPC segmentation results across diverse tumor volumes (ranging from 0.6 to 21.9 $cm^{3}$ ). The ground truth is rendered in green, and the predicted tumor surface generated by MambaGLA-NPC is overlaid in red. Spatial orientations are indicated by S (Superior), I (Inferior), A (Anterior), P (Posterior), and L (Left).

### 4.3 Combined scanning strategy performance

In our proposed MambaGLA-NPC architecture, we introduce a novel scanning strategy by combining local scanning with vertical scanning, along with their respective reverse processes. We hypothesize that vertical scanning captures long-range dependencies along anatomically dominant directions, while local scanning dynamically enhances feature representation near tumor boundaries. The combination of these approaches overcomes the limitations of existing methods in balancing global receptive fields with local accuracy. To validate this strategy, we conducted systematic ablation experiments by individually and jointly varying the scanning directions, including horizontal, vertical, cross, and local scanning, while keeping all other model components fixed.

As shown in Table 4, vertical scanning alone achieves exceptionally high recall, significantly outperforming horizontal scanning. This demonstrates its inherent advantage in capturing critical target regions within such anatomical structures. The introduction of local scanning further compensates for vertical scanning’s shortcomings in fine-grained feature modeling, creating effective functional complementarity. However, the composite strategy combining local, horizontal, and vertical scanning did not yield the expected performance boost; instead, it showed declines in multiple metrics. This indicates potential representation overlap or interference between different scanning modes, demonstrating that simple stacking does not necessarily lead to benefits. It is also notably worth mentioning that while the optimal strategy did not achieve the peak recall rate of single vertical scanning, it achieved a better balance between overall segmentation accuracy and boundary localization quality. This will be more beneficial for precisely delineating the target area, providing a foundation for reducing damage to normal tissue.

**TABLE 4:** Performance of different scanning strategies in NPC tasks.

Scanning strategy	Dice (%)	IOU(%)	Recall (%)	HD95 (mm)
Horizontal Scanning	78.42	65.03	82.51	4.88
Vertical Scanning	78.44	65.05	**84.41**	4.98
Cross Scanning	78.04	64.57	78.02	5.23
Local Scanning	78.89	65.67	82.21	**4.62**
Local Scanning + Horizontal Scanning	78.94	65.89	80.78	5.05
Local Scanning + Vertical Scanning	**79.43**	**66.37**	80.79	**4.62**
Local Scanning + Horizontal Scanning + Vertical Scanning	79.05	66.00	79.28	5.15

The best result among the compared scanning strategies is highlighted in bold.

### 4.4 NPC-adapter capability

To investigate performance variations across different clinical imaging environments, we first trained and evaluated the baseline MambaGLA-NPC model independently on each center’s dataset. As illustrated in Figure 9, performance discrepancies exist among centers, highlighting the substantial data heterogeneity introduced by distinct scanner manufacturers and imaging protocols.

**FIGURE 9:**
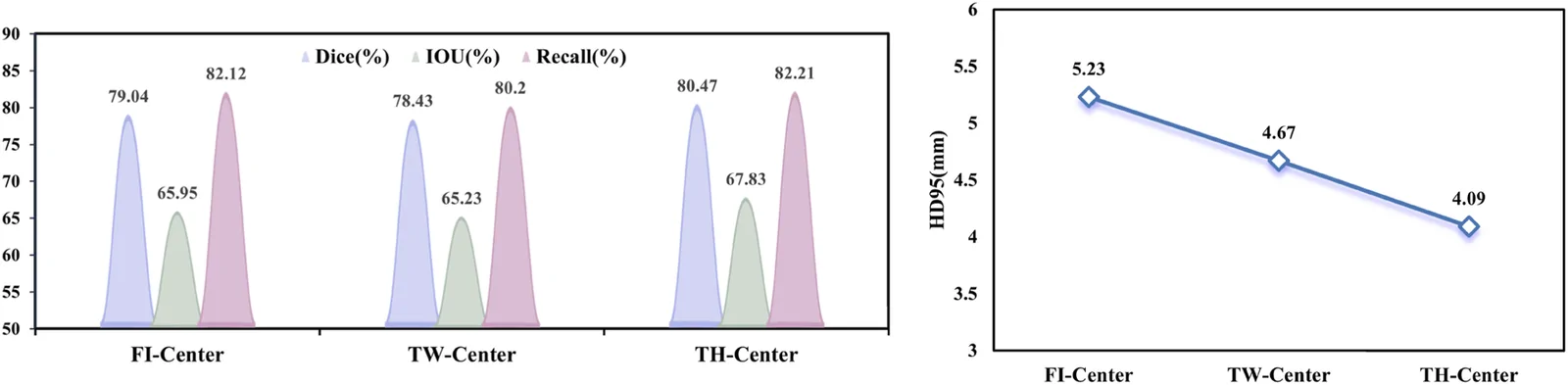
Quantitative performance comparison across different medical centers using baseline MambaGLA-NPC, demonstrating the domain gap across multi-center datasets.

To address these domain shifts and evaluate model transferability, we conducted a comprehensive evaluation of the proposed NPC-Adapter across both intra-center and cross-center scenarios, as presented in Table 5. For intra-center evaluation, models were trained and tested within the same institution to compare the baseline without adapters against the architecture integrated with the NPC-Adapter. For cross-center transfer tasks across all source-to-target pairs, two strategies were examined: direct zero-shot inference (No Adapter), where the source pre-trained model was directly evaluated on the unseen target center’s test set without any weight updates, and NPC-Adapter. In the adaptation setting, all backbone weights pre-trained on the source domain were completely frozen to retain generalized anatomical representations, and only the lightweight adapters were fine-tuned using the complete training split of the target center (i.e., n=88 for FI-Center, n=390 for TW-Center, and n=509 for TH-Center). The adapted models were subsequently evaluated on the corresponding target center’s independent test set ( n=10 for FI-Center, n=44 for TW-Center, and n=57 for TH-Center).

**TABLE 5:** Domain adaptation performance of NPC-adapter in cross-center and intra-center NPC segmentation tasks.

Base dataset	Adaptation setting	Dice (%)	IOU(%)	HD95 (mm)	Recall (%)
**FI-Center**	FI-Center → FI-Center (No Adapter) †	78.46	65.38	5.51	81.55
​	**FI-Center** → **FI-Center (NPC-Adapter)** †	**79.04**	**65.95**	**5.23**	**82.12**
​	FI-Center → TW-Center (No Adapter)	68.52	53.17	10.24	**84.10**
​	**FI-Center** → **TW-Center (NPC-Adapter)**	**75.05**	**60.73**	**7.07**	78.85
​	FI-Center → TH-Center (No Adapter)	66.83	51.61	11.07	**83.07**
​	**FI-Center** → **TH-Center (NPC-Adapter)**	**75.67**	**61.50**	**6.23**	78.76
**TW-Center**	TW-Center → TW-Center (No Adapter) †	78.15	64.88	4.96	79.58
​	**TW-Center** → **TW-Center (NPC-Adapter)** †	**78.43**	**65.23**	**4.67**	**80.20**
​	TW-Center → FI-Center (No Adapter)	74.52	60.15	7.75	75.30
​	**TW-Center** → **FI-Center (NPC-Adapter)**	**76.31**	**62.33**	**7.15**	**77.99**
​	TW-Center → TH-Center (No Adapter)	80.79	68.22	3.96	81.14
​	**TW-Center** → **TH-Center (NPC-Adapter)**	**81.15**	**68.69**	**3.79**	**81.58**
**TH-Center**	TH-Center → TH-Center (No Adapter) †	79.98	67.42	4.38	81.60
​	**TH-Center** → **TH-Center (NPC-Adapter)** †	**80.47**	**67.83**	**4.09**	**82.21**
​	TH-Center → FI-Center (No Adapter)	76.70	63.04	6.60	78.04
​	**TH-Center** → **FI-Center (NPC-Adapter)**	**78.58**	**65.40**	**6.42**	**80.32**
​	TH-Center → TW-Center (No Adapter)	87.13	77.46	2.48	**88.21**
​	**TH-Center** → **TW-Center (NPC-Adapter)**	**87.22**	**77.61**	**2.27**	88.13

The best result within each source-target adaptation pair is highlighted in bold.

Quantitative results in Table 5 demonstrate that incorporating the NPC-Adapter consistently enhances segmentation accuracy and boundary alignment across virtually all intra-center and cross-center settings. Within individual centers, the NPC-Adapter steadily refines performance (e.g., improving Dice from 78.46% to 79.04% on FI-Center and from 79.98% to 80.47% on TH-Center), confirming that the dual-adapter design effectively enriches local-global feature interactions even without cross-scanner domain shifts. In cross-center transfer scenarios, direct inference without adaptation experiences substantial performance drops due to severe scanner discrepancies (e.g., Dice drops to 68.52% and 66.83% when transferring from FI-Center to TW-Center and TH-Center, respectively). In contrast, fine-tuning the NPC-Adapter with target training data markedly boosts transfer Dice scores to 75.05% (+6.53%) and 75.67% (+8.84%), while dramatically reducing HD95 from 10.24 mm to 7.07 mm and from 11.07 mm to 6.23 mm, respectively. Furthermore, when transferring between domains with closer feature distributions (e.g., TW-Center → TH-Center) or when the source model already exhibits strong baseline performance (e.g., TH-Center → TW-Center, baseline Dice 87.13%), the NPC-Adapter continues to yield consistent improvements, particularly refining boundary delineation metrics like HD95 from 2.48 mm down to 2.27 mm.

Crucially, the NPC-Adapter introduces only 0.28M trainable parameters, representing approximately 3.7% of the full model’s 7.6M parameters. This parameter-efficient design allows rapid convergence on target training data while effectively preventing catastrophic forgetting and overfitting typically encountered in full-parameter fine-tuning.

### 4.5 Ablation study

#### 4.5.1 The effectiveness of proposed modules

To validate the effectiveness of each core component in the proposed model, we conducted systematic ablation experiments, with results shown in Table 6. The experimental results demonstrate that the comprehensive model achieves the optimal performance across all three core metrics—Dice, IoU, and HD95—while also attaining a competitive second-best level in Recall. This fully demonstrates the rationality and effectiveness of the overall architectural design.

**TABLE 6:** Ablation Study for Different Modules on the TH-Center Test Set. The best result is highlighted in bold, and the second-best result is underlined.

Methods	Dice (%)	IOU(%)	HD95 (mm)	Recall (%)
**Ours w/o GLSSM**	79.29	66.22	4.87	**82.69**
**Ours w/o CNN**	77.50	63.81	5.31	81.74
**Ours w/o BiAtt**	79.16	66.04	4.59	79.97
**Ours w/o GCS Att**	79.55	66.61	4.46	81.34
**Ours**	**80.47**	**67.83**	**4.09**	82.21

Specifically, ablating the dual-branch encoder components reveals that both local CNN features and global SSM context are indispensable: removing the CNN branch leads to the most significant degradation (Dice drops by 2.97%–77.50%, and HD95 worsens to 5.31 mm), while removing the GLSSM branch notably harms boundary precision (HD95 increases to 4.87 mm). Furthermore, removing the BiAtt module leads to a 1.31% decrease in Dice and a 1.79% decrease in IoU, while removing the GCS Att module results in a 0.92% Dice and 1.22% IoU decline. Although these volume-overlap variations appear modest in absolute percentage, their consistency across both Dice and IoU confirms non-trivial gains within this high-performance regime.

More importantly, the proposed attention modules exhibit pronounced contributions to boundary localization, with the HD95 deteriorating from 4.09 mm in the full model to 4.59 mm (w/o BiAtt) and 4.46 mm (w/o GCS Att). In clinical NPC radiotherapy planning, millimeter-level boundary accuracy is paramount because lesions are intimately surrounded by critical radiosensitive organs-at-risk (e.g., brainstem, spinal cord, and optic chiasm). By adaptively suppressing non-tumor background noise along skip connections via GCS Att and resolving feature discrepancies between the CNN and SSM branches via BiAtt, our complete framework achieves significantly tighter 3D boundary fidelity and morphological reliability.

#### 4.5.2 The high efficiency of MambaGLA-NPC

We validated the efficiency of the MambaGLA-NPC architecture through ablation experiments. As shown in Table 7, we replaced the core module in the feature extractor and compared it with the following baseline methods: Large-Kernel Convolutions used in 3D UXNet (Lee et al., 2022), the Swin Transformer relied upon by Swin UNETR (Hatamizadeh et al., 2022a), and the classic Self-Attention mechanism.

**TABLE 7:** Presents ablation experiment results for different global modeling modules, including Large-Kernel Convolutions, Swin Transformer, Self-Attention mechanisms, and the Conv-GLSSM module proposed in this paper. Experiments were conducted at an input resolution of $128^{3}$ and sequence length of 262144, comparing training memory (TM), inference memory (IM), number of parameters (Params), and computational cost (FLOPs) across modules. Values marked with^*^ are theoretical estimates.

Core	Input	Sequence	TM(M)	IM(M)	Params(M)	FLOPs(G)	Is
Module	Resolution	Length	​	​	​	​	Global
**Large-Kernel Convolution**	$128^{3}$	262144	18084	**5436**	**0.52**	26.81	✗
**Swin Transformer**	$128^{3}$	262144	20578	5976	4.92	51.99	✗
**Self-Attention**	$128^{3}$	262144	OOM	$262144^{*}$	4.92^*^	—	✓
**Conv-GLSSM**	$128^{3}$	262144	**17222**	6064	1.19	**21.66**	✓

The best performance across the compared core modules is highlighted in bold.

Although large-kernel convolutions and Swin Transformers both aim to expand the receptive field to capture long-range dependencies, their modeling capabilities face inherent limitations. Specifically, the global perception of large-kernel convolutions is constrained by their fixed kernel size. While Swin Transformers establish global contextual connections through a sliding window mechanism, their attention computations are limited to local windows. Cross-window information exchange remains indirect and gradual, falling short of true global perception. In contrast, while the classical self-attention module possesses theoretical global modeling capabilities, its computational complexity grows quadratically with sequence length. This leads to prohibitive computational overhead when processing high-resolution 3D data (e.g., sequences up to 260k in length), resulting in memory overflow and resulting in impracticality.

By comparison, our approach introduces the Mamba-based Conv-GLSSM module. This module inherits the linear complexity advantage of SSM for long sequence modeling, enabling efficient capture of global dependencies. Simultaneously, its integrated convolutional operations effectively enhance local feature extraction. Thanks to this design, our model achieves the best performance in training memory and FLOPs metrics even at extreme sequence lengths, while demonstrating comparable effectiveness across other metrics.

## 5 Discussion

In clinical practice, NPC lesions exhibit extensive volumetric variations, ranging from occult micro-lesions to bulky invasive masses, making scale-invariant segmentation a crucial metric for radiotherapy contouring. To investigate potential size-dependent performance degradation, we quantitatively evaluated the Dice accuracy as a function of ground-truth tumor volume via LOWESS regression and stratified subgroup analysis as shown in Figure 10. Overall, the segmentation accuracy showed no significant correlation with tumor volume (Pearson r=0.0718 , p=0.4417 ). Although visual segmentation in complex boundaries can appear more challenging in small lesions, the LOWESS trend reveals that the Dice score quickly plateaus at approximately 80.5% beyond $4{\text{ cm}}^{3}$ . Subgroup evaluation across volume tertiles further corroborated this consistency, yielding comparable accuracy across small ( $<4.7{\text{ cm}}^{3}$ : 80.07±8.38% ), medium ( $4.7-8.9{\text{ cm}}^{3}$ : 80.61±5.77% ), and large ( $>8.9{\text{ cm}}^{3}$ : 80.53±6.66% ) cohorts. These results demonstrate that the proposed framework possesses robust size invariance and avoids performance degradation across diverse tumor spans.

**FIGURE 10:**
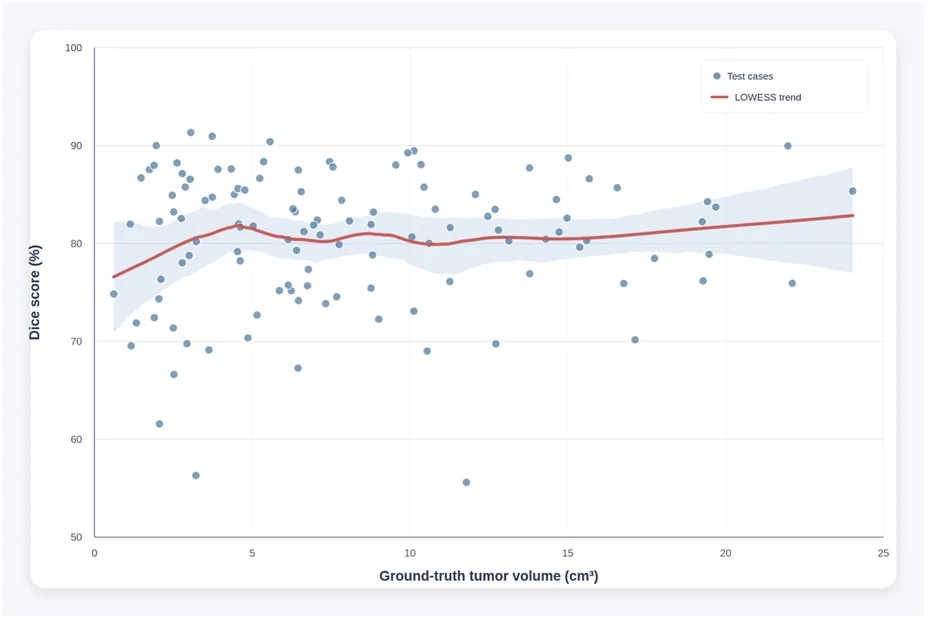
Segmentation performance by tumor volume.

This high morphological fidelity stems largely from the complementary integration of 3D CNNs and SSMs. By combining local convolutional representations with linear-complexity global state-space modeling, the Conv-GLSSM module overcomes the computational bottleneck of quadratic self-attention while maintaining sensitivity to fine boundary details. Notably, as shown in Table 4, the directional scanning ablation revealed that pairing 3D local scanning with vertical scanning achieved the best boundary localization, aligning naturally with the dominant superior-inferior anatomical axis inherent to head-and-neck volumetric scans.

Beyond architectural design, multi-center evaluation highlighted the model’s adaptability against severe domain shifts caused by heterogeneous MRI scanners and acquisition parameters. While direct cross-center inference suffered noticeable performance drops, incorporating the lightweight NPC-Adapter significantly recovered transfer performance, boosting cross-center Dice by up to 8.84% and markedly improving boundary alignment. This parameter-efficient fine-tuning strategy prevents catastrophic forgetting of general anatomical priors while enabling rapid adaptation to distinct institutional protocols.

Nevertheless, several limitations still require further investigation. The current evaluation was confined to anatomical T2 and T1C sequences, whereas the inclusion of functional modalities such as DWI and ADC could provide a more comprehensive tumor characterization. In addition, the NPC-Adapter was assessed exclusively on full target-center training sets, and its adaptability under low-data conditions therefore warrants systematic investigation. Lastly, broader clinical deployment will necessitate expanding validation to encompass a wider range of scanner field strengths, such as 1.5T.

## 6 Conclusion

In this paper, we propose MambaGLA-NPC, an MRI-based segmentation framework for NPC using Mamba. We designed a fully 3D NPC segmentation framework. Through various experiments, our framework not only outperforms existing benchmark methods in a single center but also achieves the best performance across centers. It enables global information modeling while maintaining good training and inference efficiency. Furthermore, experiments across three centers demonstrate that the proposed NPC-Adapter enables effective domain adaptation, facilitating cross-center predictions. Future work will focus on expanding model compatibility with emerging imaging protocols and exploring lightweight architectures to enhance operational flexibility without sacrificing accuracy.

## Data Availability

The datasets analyzed for this study are available from the corresponding authors upon reasonable request. The data are not publicly available due to privacy/ethical restrictions related to patient medical records.
